# The mediational role of social support in the relationship between stress and antenatal anxiety and depressive symptoms among Australian women: a mediational analysis

**DOI:** 10.1186/s12978-021-01305-6

**Published:** 2021-12-20

**Authors:** Asres Bedaso, Jon Adams, Wenbo Peng, David Sibbritt

**Affiliations:** 1 College of Medicine and Health Sciences, School of Nursing, Hawassa University, Hawassa, Ethiopia; 2 Australian Centre for Public and Population Health Research, School of Public Health, Faculty of Health, University of Technology Sydney, Ultimo, NSW Australia

**Keywords:** Stress, Depressive symptoms, Anxiety symptoms, Social support, Pregnancy, Mediation

## Abstract

**Background:**

Pregnancy can be a stressful period for most women and their family members, and the mental wellbeing of pregnant women can face serious challenges. Social support can play a role in improving the psychological well-being of pregnant women by enhancing the stress coping ability and alleviating stressful conditions. The current study aimed to assess the mediating effects of social support in the relationship between perceived stress and depressive symptoms as well as anxiety symptoms during pregnancy among Australian women.

**Methods:**

Of the 8,010 women who completed Survey 6 of the 1973–78 Australian Longitudinal Study on Women’s Health (ALSWH) cohort in 2012, those who reported being pregnant (n = 493) were included in the current analyses. Antenatal depressive and anxiety symptoms were assessed using the 10 item Center for Epidemiological Studies Depression (CES-D-10) scale, and the 9-item Goldberg Anxiety and Depression scale (GADS) respectively. The 19 item-Medical Outcomes Study Social Support index (MOSS) was used to examine social support. A parallel mediation model was used to explore the mediational role of each domain of social support between perceived stress and antenatal depressive and anxiety symptoms.

**Result:**

The study found that emotional/informational support has a partial mediating effect on the relationship between perceived stress and antenatal depressive symptoms (β = 0.371, 95% CI: 0.067, 0.799) and on the relationship between perceived stress and antenatal anxiety symptoms (β = 0.217, 95% CI: 0.029, 0.462). Affectionate support/positive social interaction and tangible support was found to play no significant mediation role between stress and antenatal depressive and anxiety symptoms.

**Conclusions:**

Emotional/informational support appears to play a mediating role in the relationship between stress and antenatal depressive as well as between stress and antenatal anxiety symptoms. In order to further protect pregnant women from the effects of stress, policy makers and maternal health professionals are advised to develop community-based social support programs to enhance prenatal psychosocial support and ensure pregnant women have adequate emotional/information support.

## Plain language summary

Social support is a resource or a means that an individual can use to cope with stressful events and improve psychological wellbeing. It improves emotional and physical well-being and promotes health for a successful pregnancy. However, the relationship between domains of social support and antenatal depressive and anxiety symptoms remains understudied in Australia. Therefore, our study intended to examine the mediating role of domains of social support in the linkage between stress and depressive and anxiety symptoms during pregnancy among Australian Women.

Data were obtained from Survey 6 of the 1973–78 ALSWH cohort, which was conducted in 2012, and those who reported being pregnant were part of the study (n = 493, aged 34–39 years). Social support provided for a pregnant woman was the outcome variable, assessed using the 19-item Medical Outcomes Study Social Support index (MOS-SSS-19 item). A parallel mediation model was used to explore the mediational role of each domain of social support between perceived stress and antenatal depressive and anxiety symptoms.

Emotional/informational support plays a mediating role in the relationship between stress and antenatal depressive as well as between stress and antenatal anxiety symptoms. So, to further protect pregnant women from the effect of stress, policymakers and maternal health professionals are advised to develop community-based social support programs.

## Background

Pregnancy is accompanied by changes to a woman’s body hormones, physical appearance, lifestyle, roles and responsibilities [1, 2]. Such changes can cause stress in pregnant women [3] and lead to an increased risk of developing mental health problems such as depressive symptoms [2], and anxiety symptoms [4].

Depression and anxiety are among the most prevalent mental health problems experienced by pregnant women [5, 6]. An estimated prevalence of antenatal depression reported by studies conducted in Australia ranges from 6–7% [7, 8] to 16.9% [9], while the prevalence of antenatal anxiety in Australia ranges from 14–59% [10–15]. Depression and anxiety during pregnancy adversely affect several obstetric and foetal outcomes and cause an increased rate of pregnancy complications and postnatal mental health problems [16–19]. Untreated antenatal anxiety and depression may lead to postnatal depression for the mother which may also result in an impaired interaction with her infant [20–22].

Social support is a resource or a means that an individual can use to cope with stressful events and improve psychological wellbeing [23]. It is defined as the provision of emotional, informational, affectionate, and tangible (i.e. financial or instrumental) support for somebody by the available social network (i.e. family members, friends, and/or community members) [24]. Social support can strengthen social relationships and promotes health and well-being for a successful pregnancy [25].

Different hypotheses have suggested several mechanisms of action of social support in preventing prenatal mental health problems. First, social support plays a stress-buffering role which directly contributes to the well-being of individuals by enhancing positive affect and/or perceived self-worth of individuals and indirectly improves well-being by alleviating stressful conditions [26]. Second, per the psycho-neuroimmunology (PNI) framework [27], social support can change negative responses related to stress, which help individuals to improve their problem-solving skill and develop a positive view about themselves[28, 29]. Third, the behavioural mechanism approach also considered social support as the support needed during a stressful event to enhance the stress coping ability, which in turn reduces the risk of mental illness [30]. The psychosocial stress hypothesis suggested social support as a preventive factor to reduce the risk of prenatal depression [31] and anxiety [32] and depressive symptoms in the general population [33–35].

The stress-buffering hypothesis supports the mediating role of social support in the linkage between stress and antenatal depressive and antenatal anxiety symptoms, which hypothesizes that social support can protect people facing stress from developing mental health problems, such as depression and anxiety [23]. This mediating effect may change an individual's perceptions about undesirable events, and provide solutions by encouraging changes in an individual's adaptive responses [36] and assist people in getting the skills required to buffer the effects of stressors [37], subsequently, the occurrence of adverse consequences will be less likely [38].

Although few studies have identified that overall social support has a mediating effect on the linkage between stress and risk of developing mental illness during pregnancy [39, 40], the linkage between specific domains of social support (emotional/informational support, affectionate support and tangible support) and depressive and anxiety symptoms during pregnancy needs further investigation among pregnant women. In response, the study reported here aimed to directly fill this knowledge gap by examining the mediating role of emotional/informational support, affectionate support/positive social interaction and tangible support in the linkage between stress and depressive and anxiety symptoms among pregnant Australian Women using nationally representative secondary data from the 1973–78 ALSWH cohort.

## Methods

### Study design and data source

This study used data from the 1973–78 cohort of the Australian Longitudinal Study on Women’s Health (ALSWH) [41, 42]. The ALSWH is an ongoing nationally representative community-based longitudinal study focusing on the health and well-being of Australian women. Over 40,000 women were recruited to participate in 1996 (baseline) in three age cohorts (birth year: 1973–78, 1946–51 and 1921–26). Participants were selected randomly via the national health insurance database (Medicare) and asked to complete mailed surveys every 3 years on average. Of the 8,010 women who completed Survey 6 of the 1973–78 cohort in 2012 (age between 34–39 years), those who reported being pregnant (n = 493) were included in the current analyses [43].

### Measurement

Depression was assessed using the 10-item Center for Epidemiological Studies Depression (CES-D-10) scale and has good reliability (α = 0.79) [44]. Items were summed to form a total score, ranging from 0 to 30, with higher scores indicating a greater level of depressive symptoms. The CES-D-10 has been used to examine depressive symptoms during pregnancy with good reliability and validity [45–49]. Anxiety symptoms were assessed using the 9-item anxiety subscale of the Goldberg Anxiety and Depression scale (GADS). Items were summed to form a total score, ranging from 0 to 9, with higher scores indicating a greater level of anxiety symptoms. The scale has good reliability (α = 0.77) [50].

The Medical Outcomes Study Social Support index (MOS-SSS-19) was used to examine social support given to pregnant women. The MOS-SSS-19 has an overall index of 19 items (Cronbach’s alpha 0.81), with higher scores indicating greater social support. The MOS-SSS-19 has three functional support subscales: emotional/informational support, tangible support, affectionate support/positive social interaction [51]. The level of stress in the last 12 months among study participants was assessed using the Perceived Stress Questionnaire, which has been developed and validated for the ALSWH study [52]. The tool examined the level of perceived stress in specific areas of life, including study, relationships and own health. An overall mean stress score was determined, which ranges from 0 (no stress) to 4 (extreme stress). The Perceived Stress Questionnaire has good internal reliability (α = 0.75) [53, 54].

### Mediation model

**Fig. 1:**
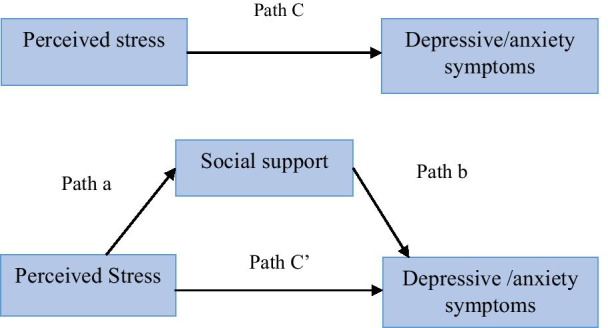
The stress-buffering model as the main hypothesis of this study

This study used the stress-buffering hypothesis [26] to explore the mediational role of social support in the relationship between perceived stress, domains of social support and prenatal depressive or anxiety symptoms (Fig. 1). The stress-buffering hypothesis suggests that social support directly contributed to the well-being of individuals by enhancing positive affect and perceived self-worth (main effect). However, social support may also indirectly improve the well-being of individuals by alleviating stressful conditions or by reducing the impacts of stressful situations (buffering effect) [26].

During the application of the stress-buffering hypothesis, we expected that pregnant mothers with increased levels of stress would have a higher risk of depressive or anxiety symptoms. In contrast, it is expected that pregnant women with high social support would have less risk of antenatal depressive or anxiety symptoms. Finally, we hypothesized that social support would mediate or intervene in the effects of levels of stress on antenatal depressive or antenatal anxiety symptoms.

### Data analysis

The statistical software package SPSS Statistics 26.0 was used for all analyses. The one-way ANOVA and independent-sample t-test were used to examine group mean differences of continuous variables. In addition, an initial correlational analysis was used to test the relationships between stress, domains of social support, and antenatal depressive symptoms and antenatal anxiety symptoms.

The mediational role of social support between perceived stress and antenatal depressive and anxiety symptoms was examined using a mediational analysis model which is conducted using PROCESS macro (version 3.0) for SPSS. For a variable to be considered as a mediator, it should fulfil the following criteria: (1) the independent variables (stress) should have a strong association with dependent variables (anxiety and/or depression); (2) the independent variables should be strongly related to a mediator (emotional/informational support, affectionate support and tangible support); and (3) independent variables and mediator should be related to outcome variables. However, if the independent variable is no longer significant when the mediator variable is controlled, the finding will be a full mediation effect. If the independent variable still shows significant association when the mediator is controlled, the finding can be considered as a partial mediation effect [55].

Therefore, a 3-step analysis was performed to test the mediating effects of social support in the relationship between stress and antenatal depressive symptoms and antenatal anxiety symptoms. In the first step, each domain of social support is regressed on stress. In the second step, antenatal depressive symptoms and antenatal anxiety symptoms regressed on stress separately. In the third step, the outcome variables (antenatal depressive symptoms and antenatal anxiety symptoms) are regressed on stress and domains of social support separately. The total effect (path c), indirect effects (path a*b) and direct effects (path c’) were reported in the form of unstandardized beta coefficients ($\upbeta$). The bootstrapping procedures in the SPSS PROCESS macro from the parallel mediation model 4 were used to test the significance of the indirect effects of stress on antenatal depressive symptoms and antenatal anxiety symptoms through the mediation of each domain of social support [56]. The mediation effect is significant (p < 0.05) if the 95% confidence interval (CI) for the result of the mediation effect did not contain zero. During the analysis, multicollinearity is not considered a problem if the Variance inflation factor (VIF) values are less than 5 [57].

## Result

Demographic characteristics of pregnant women and group mean differences in stress, social support, depressive and anxiety symptoms are shown in Table 1. The mean (Standard Deviation) age of the participants was 35.8 years (1.4) and the majority of participants (65.7%) were between the age of 34–36 years, (95.1%) were married/in a de facto relationship, while (65%) achieved a university degree. The majority of the women (42%) were in the last trimester of their pregnancy, while 37.5% and 20.5% were in the second and first trimester respectively.

**Table 1:** Relationship between demographic characteristics and stress, social support as well as antenatal depressive and anxiety symptoms among Australian women, 2021

Variables	n (%)	Stress (mean ± SD)	Social support (mean ± SD)			Anxiety symptoms (mean ± SD)	Depressive symptoms (mean ± SD)
			Emotional support	Affectionate support	Tangible support		
Age							
34–36	324 (65.7)	0.57 (0.397)	4.41 (0.735)	4.57 (0.586)	4.29 (0.798)	3.43 (2.447)	5.23 (4.262)
37–39	169 (34.3)	0.68 (0.471)	4.23 (0.868)	4.36 (0.776)	4.09 (0.857)	3.43 (2.288)	5.78 (4.278)
*P*-value		0.008	0.022	0.001	0.012	0.994	0.178
Stage of pregnancy							
< 3 month	101 (20.5)	0.51 (0.386)	4.41 (0.735)	4.52 (0.611)	4.25 (0.799)	3.40 (2.350)	5.23 (0.897)
3–6 month	185 (37.5)	0.64 (0.424)	4.27 (0.840)	4.44 (0.695)	4.19 (0.856)	3.54 (2.512)	5.30 (4.466)
> 6 month	207 (42)	0.442 (0.031)	4.38 (0.759)	4.54 (0.662)	4.23 (0.808)	3.34 (2.307)	5.63 (4.267)
*P-*value		0.042	0.266	0.367	0.816	0.717	0.664
Highest qualification							
University	319 (65)	0.60 (0.412)	4.38 (0.752)	4.53 (0.646)	4.23 (0.826)	3.33 (2.321)	5.19 (4.071)
Certificate/diploma or trade/apprenticeship	112 (22.8)	0.65 (0.452)	4.29 (0.835)	4.47 (0.698)	4.26 (0.749)	3.69 (2.479)	5.76 (4.652)
School only	60 (12.2)	0.58 (0.457)	4.26 (0.873)	4.39 (0.698)	4.09 (0.934)	3.52 (2.561)	5.99 (4.576)
*P*-value		0.492	0.439	0.298	0.383	0.389	0.268
Marital status							
Married/De facto relationship	468 (95.1)	0.60 (0.426)	4.38 (0.765)	4.52 (0.649)	4.25 (0.802)	3.43 (2.396)	5.34 (4.222)
Divorced/single/separated	24 (4.9)	0.74 (0.426)	3.78 (0.990)	3.99 (0.770)	3.59 (0.989)	3.50 (2.207)	7.06 (4.945)
*P*-value		0.108	< 0.001	< 0.001	< 0.001	0.893	0.054
Able to manage on income available							
Impossible/Difficult all of the time	43 (8.8)	1.06 (0.612)	3.57 (1.215)	3.91 (0.931)	3.60 (1.059)	5.10 (2.424)	9.42 (5.406)
Difficult some of the time	118 (24)	0.69 (0.416)	4.35 (0.715)	4.50 (0.602)	4.18 (0.870)	3.76 (2.367)	5.95 (4.632)
Not too bad/It is easy	330 (67.2)	0.52 (0.353)	4.45 (0.680)	4.57 (0.606)	4.32 (0.733)	3.09 (2.278)	4.70 (3.625)
*P*-value		< 0.001	< 0.001	< 0.001	< 0.001	< 0.001	< 0.001

*SD* Standard deviation

Marital status was found to be significantly related with domains of social support, and pregnant women who are married/in a de facto relationship reported a higher score of emotional/informational (p < 0.001), affectionate (p < 0.001) and tangible support (p < 0.001) than those with divorced/single/separated marital status. Also, pregnant women who can easily manage on income available presented less stress level (p < 0.001), less depressive (p < 0.001) and anxiety symptoms (p < 0.001) and a higher score of emotional (p < 0.001), affectionate (p < 0.001) and tangible support (p < 0.001).

### Correlations among continuous variables

Table 2 presents the results of the correlation analysis. Prior to conducting mediational analysis, it is necessary to check whether the independent, mediating and dependent variables are correlated with each other. Perceived stress was negatively related to emotional/informational support (*r* = − 0.398, p < 0.001), affectionate support (*r* = − 0.433, p < 0.001) and tangible support (*r* = − 0.321, p < 0.001), and positively related with depressive (*r* = 0.557, p < 0.001), and anxiety symptoms (*r* = 0.560, p < 0.001). Depressive symptoms were negatively related to emotional/informational support (r = − 0.471, p < 0.001), affectionate support (*r* = − 0.454, p < 0.001) and tangible support (*r* = − 0.359, p < 0.001). Similarly, anxiety symptoms were negatively related to emotional/informational support (*r* = − 0.369, p < 0.001), affectionate support (*r* = − 0.359, p < 0.001) and tangible support (*r* = − 0.289, p < 0.001). These bivariate correlations support the following mediation analyses.

**Table 2:** Correlations between age, stress, domains of social support, antenatal depressive symptoms and antenatal anxiety symptoms among Australian women, 2021

S.no		Mean ± SD	1	2	3	4	5	6
1	Age	35.8 (± 1.4)	1					
2	Perceived Stress	0.61 (0.42)	0.067	2				
3	Antenatal depression symptoms	5.42 (4.27)	0.062	0.557**	3			
4	Antenatal anxiety symptoms	3.43 (2.39)	− 0.002	0.560**	0.665**	4		
5	Emotional/Informational support	4.35 (0.78)	− 0.119**	− 0.398**	− 0.471**	− 0.369**	5	
6	Affectionate support/Positive social interaction	4.50 (0.66)	− 0.168**	− 0.433**	− 0.454**	− 0.359**	0.828**	6
7	Tangible support	4.22 (0.82)	− 0.139**	− 0.321**	− 0.359**	− 0.289**	0.676**	0.673**

**Correlation is significant at the p < 0.01 level (2-tailed)

### The mediational role of social support

**Fig. 2:**
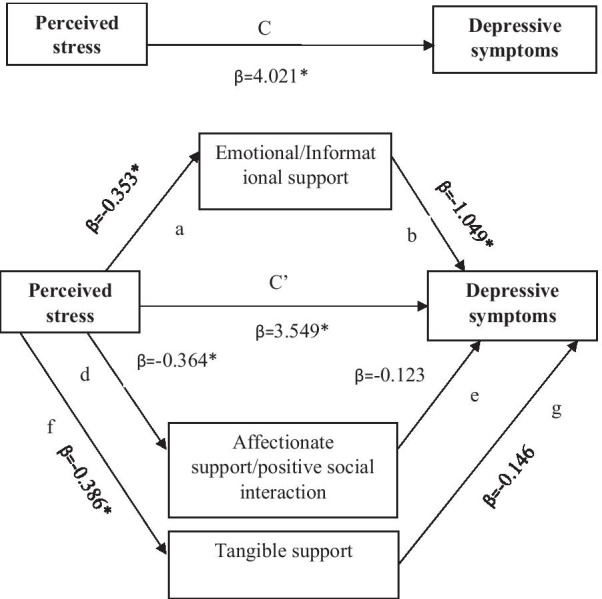
Model of the mediating role of domains of social support between perceived stress and depressive symptoms. **p < 0.01

The first mediational analysis was performed to examine the mediational role of social support on the linkage between stress and antenatal depressive symptoms. The results presents in Table 3 show that the total effect of stress on antenatal depressive symptoms was statistically significant (β = 4.021, p < 0.001). With the inclusion of the mediating variables (emotional support/informational support, affectionate support/positive social interaction and tangible support), the effect of stress on antenatal depressive symptoms reduced but remained statistically significant (β = 3.549, p < 0.001). The indirect effect of perceived stress on antenatal depressive symptoms through affectionate support/positive social interaction (β = 0.044, 95% CI: − 0.325, 0.405) and tangible support (β = 0.056, 95% CI: − 0.142, 0.274) was statistically non-significant. However, the indirect effect of perceived stress on antenatal depressive symptoms through emotional/informational social support was found to be statistically significant (β = 0.371, 95% CI: 0.067, 0.799). This implies the relationship between perceived stress and antenatal depressive symptoms is partially mediated by emotional/informational support. The display of the parallel mediation model was presented in Fig. 2.

**Table 3:** Bootstrapping indirect effects and 95% confidence intervals (CI) for the mediational analysis in the relationship between perceived stress and antenatal depressive and anxiety symptoms among Australian women, 2021

Effect	SE	β coefficient (effect)	*P*-value	95% CI
Indirect effect (a*b)				
Perceived stress → Emotional/informational support → depressive symptoms^‡^	0.189	0.371	–	(0.067, 0.799)*
Perceived tress → Affectionate support/positive social interaction → depressive symptoms^‡^	0.184	0.044	–	(− 0.325, 0.405)
Perceived tress → Tangible support → depressive symptoms^‡^	0.102	0.056	–	(− 0.142, 0.274)
Perceived tress → emotinal/informational support → anxiety symptoms^¥^	0.113	0.217	–	(0.029, 0.462)*
Perceived tress → affectionate support/positive social interaction → anxiety symptoms^¥^	0.109	− 0.012	–	(− 0.239, 0.198)
Perceived tress → tangible support → anxiety symptoms^¥^	0.067	0.053	–	(− 0.079, 0.194)
Direct effect (c’) s				
Perceived tress → depressive symptoms	0.428	3.549	< 0.001	(2.708, 4.391)*
Perceived tress → anxiety symptoms	0.246	2.688	< 0.001	(2.204, 3.172)*
Total effect (c)				
Perceived tress → depressive symptoms	0.428	4.021	< 0.001	(3.180, 4.863)*
Perceived tress → anxiety symptoms	0.239	2.947	< 0.001	(2.477, 3.417)*

^‡^Model adjusted for sociodemographic factors (Age, marital status), stage of pregnancy, and history of miscarriage, life satisfaction and optimism

^¥^Model adjusted for sociodemographic factors (Age, marital status), stage of pregnancy, history of miscarriage, and life satisfaction

*P** < **0.001

**Fig. 3:**
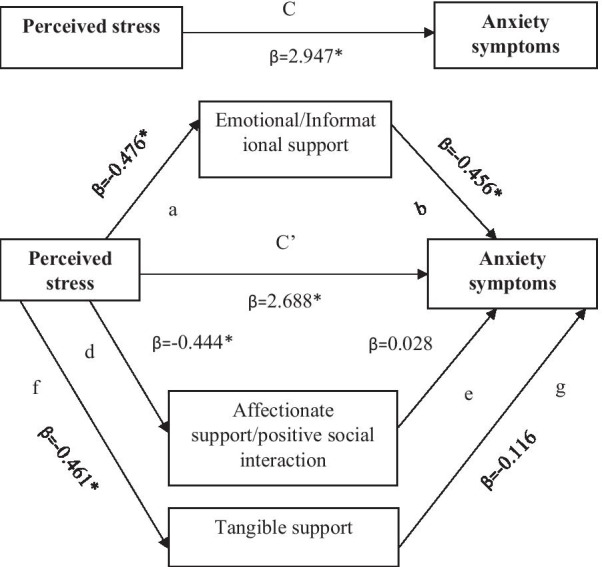
Model of the mediating role of domains of social support between perceived stress and anxiety symptoms. **p < 0.01

Similarly, a mediational analysis was performed to examine the mediational role of social support on the relationship between perceived stress and antenatal anxiety symptoms. The results revealed that the total effect of perceived stress on antenatal anxiety symptoms was statistically significant (β = 2.947, p < 0.001) (Table 3). With the inclusion of the mediating variables (emotional support/informational support, affectionate support/positive social interaction and tangible support), the effect of perceived stress on antenatal anxiety symptoms was slightly reduced but remained statistically significant (β = 2.688, p < 0.001). The indirect effect of perceived stress on antenatal anxiety symptoms through emotional/informational social support was statistically significant (β = 0.217, 95% CI: 0.029, 0.462). Therefore, the finding demonstrated that the relationship between perceived stress and antenatal anxiety symptoms is partially mediated by emotional/informational support. However, the indirect effect of perceived stress on antenatal anxiety symptoms through affectionate support/positive social interaction (β = − 0.012, 95% CI: − 0.239, 0.198) and tangible support (β = 0.053, 95% CI: − 0.079, 0.194)) was found to be not statistically significant. The display of the parallel mediation model was presented in Fig. 3.

## Discussion

This study aimed to examine the mediating effects of domains of social support in the relationship between stress and depressive and anxiety symptoms during pregnancy among Australian women, demonstrating a number of important findings. This study supplements limited evidence investigating the mediating role of domains of social support in the relationship between perceived stress and antenatal depressive and anxiety symptoms. In fact, this is the first study to examine the mediating effect of specific domains of social support in the linkage between perceived stress and antenatal depressive and antenatal anxiety symptoms among Australian women.

Our study shows that emotional/informational support has a significant partial mediational role in the relationship between stress and antenatal depressive and anxiety symptoms. Conversely, we also found that affectionate support/positive social interaction and tangible support have no significant mediation role in the link between stress and antenatal depressive and antenatal anxiety symptoms.

Two previous studies have assessed the mediational role of overall social support between stress and depressive symptoms [58, 59] as well as anxiety symptoms [58] among pregnant women. The first was a community-based study conducted among a sample of 755 pregnant Chinese women to investigate the roles of social support in assisting the stress coping ability of pregnant women with depressive and anxiety symptoms. The study found that subjective, objective and total social support each plays a significant direct effect on prenatal depression. Besides, this study indicated that social support has a mediating effect in improving prenatal depression and anxiety [58]. The other study conducted in Gondar, Ethiopia (n = 916), have shown that partner and social support partially mediated the association between stressors and antenatal depression [59]. In Australia, an organization known as PANDA (Perinatal Anxiety and Depression Australia) has offered nationwide telephone-based helpline support provided by counsellors for pregnant women, and their families experiencing mental health problems which played a significant role for women to recover from perinatal mental illness (http://www.panda.org.au). There is mixed evidence on the effectiveness of telephone support. A randomized control trial (RCT) conducted on assessing the effectiveness of a telephone support program for pregnant women in New Zealand found the intervention group at 34 weeks of gestation reported less stress, anxiety and depression levels compared to the control group [60]. Another RCT conducted in England among low risk nulliparous pregnant women indicated that telephone support by a midwife did not significantly reduce anxiety [61]. Further, a RCT conducted in Canada also found telephone support played an effective role in reducing postnatal depression [62]. In contrast, a RCT conducted in the US among pregnant women with a history of at least one spontaneous perinatal loss found that home visits by nurses did not significantly decrease anxiety levels [63].

Several mechanisms might explain our study’s identified mediational role of emotional/informational support in the linkage between perceived stress and depressive and anxiety symptoms during pregnancy. The first mechanism is the stress-buffering hypothesis, which suggests that social support directly contributed to the well-being of individuals by enhancing positive affect and/or perceived self-worth of individuals and indirectly improve well-being by alleviating stressful conditions [26]. Second, the linkage between stress, emotional support and depressive or anxiety symptoms during pregnancy can be supported by the psycho-neuroimmunology framework [27], which suggests that the roles of emotional/informational support can change negative responses related to stress, which can help individuals to improve their problem-solving skill and develop a positive view about themselves. This, in turn, can reduce the negative effect stress has on their psychological well-being and reduce the risk of depressive and/or anxiety symptoms [28, 29]. Social support gives pregnant women a better individual well-being [64] and those with a better psychosocial support tend to cope better with stressful events [23]. A strong sense of support can later give women the confidence to cope with stressful events without the help of their social network. Social support also has a significant effect on pregnant women's ability to identify possible stressors [65].

Our study found that affectionate support/positive social interaction and tangible support have no significant mediation role in the linkage between perceived stress and antenatal depressive and anxiety symptoms. These findings did not support our hypothesis that affectionate support/positive social interaction and tangible support plays a significant mediational role in the linkage between perceived stress and antenatal depressive and antenatal anxiety symptoms. One possible reason for this finding is that affectionate/positive social interaction and tangible support were measured using the MOS-SSS-19 scale, which mainly explores the perception of social support and not always reflects the actual available support in which sometimes the actual social support might not be perceived [66]. Further, the social support finding relies on self-reported data from study participants, which are potentially prone to recall bias. As a result of the above factors, the effects of the mediator variable (affectionate/positive social interaction and tangible support) will be underestimated and this might result in limited power of our analysis and false identification of a non-significant association.

Some other limitations need to be considered when making inferences from our study findings. First, the study depends on self-reported data from study participants, which has the potential to introduce recall bias. Second, our findings are limited to pregnant women within the age range of 34–39 years and as such, any interpretation of our findings with regards to other demographics and populations (including younger pregnant women) must be undertaken with caution. Studies have shown that there is variation in the level of prenatal social support across teen (15–19 years) and adult mothers (greater than 20 years) [67, 68]. The level of social support was reported to be less among teen mothers as they had less ability to make and sustain relationships with their social network [67, 68]. Despite these limitations, the significance of our study and findings is strengthened by the fact that our study provided the first analysis of data collected from a nationally representative sample of pregnant women within the age range of 34–39 years.

## Conclusion

Our study demonstrated that emotional/informational support has a partial mediating role in the relationship between perceived stress and antenatal depressive and anxiety symptoms. Our study finding suggests that emotional/informational support can play a role in helping reduce the effects of stress, which in turn can reduce the risk of depressive and/or anxiety symptoms during pregnancy. The social support provided over the course of pregnancy may change [69] and in response, there is much to be gained from conducting a longitudinal study to explore the causative relationship between social support, perceived stress, and depressive and/or anxiety symptoms over the different time periods of pregnancy. As part of routine antenatal care activity, it may be beneficial to integrate valid tools to assess for the amount and type of social support received when recording the medical history of pregnant women. In order to further protect pregnant women from the effects of stress, policymakers and maternal health professionals are advised to develop community-based social support programs to enhance prenatal psychosocial support. Such programs should also work to strengthen the social network of pregnant women and ensure pregnant women have adequate emotional/information support.

## Data Availability

After request, all analyzed data will be available from the Australian Longitudinal Study on Women’s Health (ALSWH). https://www.alswh.org.au/.

## Abbreviations

ALSWH: Australian Longitudinal Study on Women’s Health
CESD-10: 10 Item Center for Epidemiological Study Depression scale
CI: Confidence Interval
GADS: Goldberg Anxiety Depression Scale
LMICs: Low and Middle-Income Countries
LOT-R: Life Orientation Test-Revised
MOS-SSS-19: 19 Item Medical Outcome Study Social Support Scale
SD: Standard Deviation
SPSS: Statistical Package for Social Science
VIF: Variance Inflation Factor
WHO: World Health Organization

## References

[1] Guardino CM, Dunkel SC (2014). Coping during pregnancy: a systematic review and recommendations. Health Psychol Rev.

[2] Razurel C, Kaiser B, Sellenet C, Epiney M (2013). Relation between perceived stress, social support, and coping strategies and maternal well-being: a review of the literature. Women Health.

[3] Cardwell MS (2013). Stress: pregnancy considerations. Obstet Gynecol Surv.

[4] Roos A, Faure S, Lochner C, Vythilingum B, Stein D (2013). Predictors of distress and anxiety during pregnancy. Agnes Karll Schwest Krankenpfleger.

[5] Dunkel SC (2011). Psychological science on pregnancy: stress processes, biopsychosocial models, and emerging research issues. Annu Rev Psychol.

[6] Rich-Edwards JW, Kleinman K, Abrams A, Harlow BL, McLaughlin TJ, Joffe H (2006). Sociodemographic predictors of antenatal and postpartum depressive symptoms among women in a medical group practice. J Epidemiol Community Health.

[7] Ogbo FA, Eastwood J, Hendry A, Jalaludin B, Agho KE, Barnett B (2018). Determinants of antenatal depression and postnatal depression in Australia. BMC Psychiatry.

[8] Buist A, Bilszta J, Milgrom J, Condon J, Speelman C, Hayes B, et al. The beyondblue National Postnatal Depression Program, Prevention and Early Intervention 2001–2005, Final Report. Hawthorn West, Vic: beyondblue: The National Depression Initiative. 2006.

[9] Leigh B, Milgrom J (2008). Risk factors for antenatal depression, postnatal depression and parenting stress. BMC Psychiatry.

[10] Serçekuş P, Okumuş H (2009). Fears associated with childbirth among nulliparous women in Turkey. Midwifery.

[11] Rico MAG, Rodríguez AJM, Díez SMU, Real MCM (2010). Análisis de la relación entre riesgo gestacional y ansiedad materna. Progresos de Obstetricia y Ginecología.

[12] Hernández-Martínez C, Val VA, Murphy M, Busquets PC, Sans JC (2011). Relation between positive and negative maternal emotional states and obstetrical outcomes. Women Health.

[13] Nieminen K, Stephansson O, Ryding EL (2009). Women's fear of childbirth and preference for cesarean section–a cross-sectional study at various stages of pregnancy in Sweden. Acta Obstet Gynecol Scand.

[14] Teixeira C, Figueiredo B, Conde A, Pacheco A, Costa R (2009). Anxiety and depression during pregnancy in women and men. J Affect Disord.

[15] Faisal-Cury A, Menezes PR (2007). Prevalence of anxiety and depression during pregnancy in a private setting sample. Arch Womens Ment Health.

[16] Orr ST, Miller CA (1995). Maternal depressive symptoms and the risk of poor pregnancy outcome: review of the literature and preliminary findings. Epidemiol Rev.

[17] Kurki T, Hiilesmaa V, Raitasalo R, Mattila H, Ylikorkala O (2000). Depression and anxiety in early pregnancy and risk for preeclampsia. Obstet Gynecol.

[18] Freeman MP (2008). Perinatal psychiatry: risk factors, treatment data, and specific challenges for clinical researchers. J Clin Psychiatry.

[19] Hollins K (2007). Consequences of antenatal mental health problems for child health and development. Curr Opin Obstet Gynecol.

[20] Heron J, O’Connor TG, Evans J, Golding J, Glover V, Team AS (2004). The course of anxiety and depression through pregnancy and the postpartum in a community sample. J Affect Disord.

[21] Hart S, Field T, del Valle C, Pelaez-Nogueras M (1998). Depressed mothers' interactions with their one-year-old infants. Infant Behav Dev.

[22] Figueiredo B, Costa R, Pacheco A, Pais A (2009). Mother-to-infant emotional involvement at birth. Matern Child Health J.

[23] Gottlieb BH, Bergen AE (2010). Social support concepts and measures. J Psychosom Res.

[24] Cohen MM, Ansara D, Schei B, Stuckless N, Stewart DE (2004). Posttraumatic stress disorder after pregnancy, labor, and delivery. J Women's Health.

[25] Cohen S, Underwood LG, Gottlieb BH (2000). Social support measurement and intervention: a guide for health and social scientists.

[26] Cohen S, Wills TA (1985). Stress, social support, and the buffering hypothesis. Psychol Bull.

[27] McnCain NL, Gray DP, Walter JM, Robins J (2005). Implementing a comprehensive approach to the study of health dynamics using the psychoneuroimmunology paradigm. ANS Adv Nurs Sci.

[28] Ngai F-W, Chan SW-C (2012). Learned resourcefulness, social support, and perinatal depression in Chinese mothers. Nurs Res.

[29] Xia L-X, Ding C, Hollon SD, Wan L (2013). Self-supporting personality and psychological symptoms: the mediating effects of stress and social support. Personality Individ Differ.

[30] Wills TA, Ainette MC. Social networks and social support. In: Baum A, Revenson TA, Singer J, editors. Handbook of health psychology. Psychology Press; 2012. p. 465–492.

[31] Jeong H-G, Lim J-S, Lee M-S, Kim S-H, Jung I-K, Joe S-H (2013). The association of psychosocial factors and obstetric history with depression in pregnant women: focus on the role of emotional support. Gen Hosp Psychiatry.

[32] Aktan NM (2012). Social support and anxiety in pregnant and postpartum women: a secondary analysis. Clin Nurs Res.

[33] Gariepy G, Honkaniemi H, Quesnel-Vallee A (2016). Social support and protection from depression: systematic review of current findings in Western countries. Br J Psychiatry.

[34] Wang J, Mann F, Lloyd-Evans B, Ma R, Johnson S (2018). Associations between loneliness and perceived social support and outcomes of mental health problems: a systematic review. BMC Psychiatry.

[35] Santini ZI, Koyanagi A, Tyrovolas S, Mason C, Haro JM (2015). The association between social relationships and depression: a systematic review. J Affect Disord.

[36] Razurel C, Bruchon-Schweitzer M, Dupanloup A, Irion O, Epiney M (2011). Stressful events, social support and coping strategies of primiparous women during the postpartum period: a qualitative study. Midwifery.

[37] Kingston D, Heaman M, Fell D, Dzakpasu S, Chalmers B (2012). Factors associated with perceived stress and stressful life events in pregnant women: findings from the Canadian Maternity Experiences Survey. Matern Child Health J.

[38] Andreotti C, Thigpen JE, Dunn MJ, Watson K, Potts J, Reising MM (2013). Cognitive reappraisal and secondary control coping: associations with working memory, positive and negative affect, and symptoms of anxiety/depression. Anxiety Stress Coping.

[39] Lau Y, Wong DFK (2008). The role of social support in helping Chinese women with perinatal depressive symptoms cope with family conflict. J Obstet Gynecol Neonatal Nurs.

[40] Pires R, Araújo-Pedrosa A, Canavarro MC (2014). Examining the links between perceived impact of pregnancy, depressive symptoms, and quality of life during adolescent pregnancy: the buffering role of social support. Matern Child Health J.

[41] Brown WJ, Bryson L, Byles JE, Dobson AJ, Lee C, Mishra G (1999). Women's Health Australia: recruitment for a national longitudinal cohort study. Women Health.

[42] Loxton D, Tooth L, Harris ML, Forder PM, Dobson A, Powers J (2018). Cohort profile: the Australian longitudinal study on Women’s health (ALSWH) 1989–95 cohort. Int J Epidemiol.

[43] Lee C, Dobson AJ, Brown WJ, Bryson L, Byles J, Warner-Smith P (2005). Cohort profile: the Australian longitudinal study on women's health. Int J Epidemiol.

[44] Andresen EM, Malmgren JA, Carter WB, Patrick DL (1994). Screening for depression in well older adults: evaluation of a short form of the CES-D (Center for Epidemiologic Studies Depression Scale). Am J Prev Med.

[45] Canady RB, Stommel M, Holzman C (2009). Measurement properties of the centers for epidemiological studies depression scale (CES-D) in a sample of African American and non-Hispanic White pregnant women. J Nurs Meas.

[46] Mosack V, Shore ER (2006). Screening for depression among pregnant and postpartum women. J Community Health Nurs.

[47] Marcus SM, Flynn HA, Blow FC, Barry KL (2003). Depressive symptoms among pregnant women screened in obstetrics settings. J Womens Health.

[48] Holzman C, Eyster J, Tiedje LB, Roman LA, Seagull E, Rahbar MH (2006). A life course perspective on depressive symptoms in mid-pregnancy. Matern Child Health J.

[49] Seto M, Cornelius MD, Goldschmidt L, Morimoto K, Day NL (2005). Long-term effects of chronic depressive symptoms among low-income childrearing mothers. Matern Child Health J.

[50] Goldberg D, Bridges K, Duncan-Jones P, Grayson D (1988). Detecting anxiety and depression in general medical settings. BMJ.

[51] Sherbourne CD, Stewart AL (1991). The MOS social support survey. Soc Sci Med.

[52] Bell S, Lee C (2002). Development of the perceived stress questionnaire for young women. Psychol Health Med.

[53] Bell S, Lee C (2003). Perceived stress revisited: the Women's Health Australia project young cohort. Psychol Health Med.

[54] Bell S, Lee C (2006). Does timing and sequencing of transitions to adulthood make a difference? Stress, smoking, and physical activity among young Australian women. Int J Behav Med.

[55] Baron RM, Kenny DA (1986). The moderator–mediator variable distinction in social psychological research: conceptual, strategic, and statistical considerations. J Pers Soc Psychol.

[56] Hayes AF (2012). PROCESS: A versatile computational tool for observed variable mediation, moderation, and conditional process modeling.

[57] O’brien RM (2007). A caution regarding rules of thumb for variance inflation factors. Qual Quant.

[58] Lau Y, Wong DFK, Wang Y, Kwong DHK, Wang Y (2014). The roles of social support in helping Chinese women with antenatal depressive and anxiety symptoms cope with perceived stress. Arch Psychiatr Nurs.

[59] Dadi AF, Miller ER, Woodman R, Bisetegn TA, Mwanri L (2020). Antenatal depression and its potential causal mechanisms among pregnant mothers in Gondar town: application of structural equation model. BMC Pregnancy Childbirth.

[60] Bullock L, Wells J, Duff G, Hornblow A (1995). Telephone support for pregnant women: outcome in late pregnancy. N Z Med J.

[61] Snaith VJ, Hewison J, Steen IN, Robson SC (2014). Antenatal telephone support intervention with and without uterine artery Doppler screening for low risk nulliparous women: a randomised controlled trial. BMC Pregnancy Childbirth.

[62] Dennis C-L, Hodnett E, Kenton L, Weston J, Zupancic J, Stewart DE (2009). Effect of peer support on prevention of postnatal depression among high risk women: multisite randomised controlled trial. BMJ.

[63] Côté-Arsenault D, Schwartz K, Krowchuk H, McCoy TP (2014). Evidence-based intervention with women pregnant after perinatal loss. MCN.

[64] Gülaçtı F (2010). The effect of perceived social support on subjective well-being. Procedia Soc Behav Sci.

[65] Nasseh Lotf Abadi M, Ghazinour M, Nojomi M, Richter J (2012). The buffering effect of social support between domestic violence and self-esteem in pregnant women in Tehran. Iran. J Fam Viol..

[66] Zanini DS, Peixoto EM (2016). Social Support Scale (MOS-SSS): analysis of the psychometric properties via item response theory. Paidéia (Ribeirão Preto).

[67] Wahn EH, Nissen E (2008). Sociodemographic background, lifestyle and psychosocial conditions of Swedish teenage mothers and their perception of health and social support during pregnancy and childbirth. Scand J Public Health.

[68] Figueiredo B, Bifulco A, Pacheco A, Costa R, Magarinho R (2006). Teenage pregnancy, attachment style, and depression: a comparison of teenage and adult pregnant women in a Portuguese series. Attach Hum Dev.

[69] Smith LE, Howard KS (2008). Continuity of paternal social support and depressive symptoms among new mothers. J Fam Psychol.

